# Supplementary material for: Effective interventions in preventing gestational diabetes mellitus: A systematic review and meta-analysis
Source: Commun Med (Lond). 2024 Apr 20;4:75. doi: 10.1038/s43856-024-00491-1 (PMC11032369; doi:10.1038/s43856-024-00491-1)
Supplement: Supplementary file 1 — Supplementary Information [file 43856_2024_491_MOESM1_ESM.pdf]

Title: Effective interventions in preventing gestational diabetes mellitus: A systematic review and meta-analysis

Wubet Worku Takele[1], Kimberly K Vesco[2], Jami Josefson[3], Leanne M Redman[4], Wesley Hannah[5,6], Maxine P Bonham[7], Mingling Chen[8], Sian C Chivers[9], Andrea J Fawcett[10,11], Jessica A. Grieger[12], Nahal Habibi[12], Gloria K.W. Leung[13], Kai Liu[13], Eskedar G Mekonnen[14], Maleesa Pathirana[12], Alejandra Quinteros[12], Rachael Taylor[15], Gebresilasea G Ukke[1], Shao J Zhou[16], ADA/EASD PMDI[*], Siew Lim[1]*

[1]Eastern Health Clinical School, Monash University, Melbourne, Victoria, Australia
[2]Kaiser Permanente Northwest, Kaiser Permanente Center for Health Research, Oakland, USA
[3]Northwestern University/ Lurie Children's Hospital of Chicago, Chicago, IL, USA
[4]Pennington Biomedical Research Center, Baton Rouge, LA, USA
[5]Madras Diabetes Research Foundation Chennai, India
[6]Deakin University, Melbourne, Australia
[7]Department of Nutrition, Dietetics and Food, Monash University, Melbourne, Victoria, Australia
[8]Monash Centre for Health Research and Implementation, Monash University, Clayton, Victoria, Australia
[9]Department of Women and Children's Health, King's College London, London, United Kingdom
[10]Ann & Robert H. Lurie Children's Hospital of Chicago, Chicago, IL, USA
[11]Department of Clinical & Organizational Development, University of Chicago, Chicago, IL, USA
[12]Adelaide Medical School, Faculty of Health and Medical Sciences, The University of Adelaide, Adelaide, Australia
[13]Department of Nutrition, Dietetics and Food, Monash University, Melbourne, Victoria, Australia
[14]Global Health Institute, University of Antwerp, Antwerp, Belgium
[15]School of Health Sciences, University of Newcastle, Callaghan, New South Wales, Australia
[16]School of Agriculture, Food and Wine, The University of Adelaide, Adelaide, Australia
* A list of authors and their affiliations appears at the end of the paper

***Corresponding author**

E-mail: siew.lim1@monash.edu

Mobile: +61447558686

Address: Level 2, 5 Arnold St, 3128 Box Hill, Victoria, Australia.

Supplementary tables and figures

Supplementary Table 1. Participant, Intervention, Control, and Outcome (PICO)

Supplementary Figure 1. A funnel plot showing the publication bias for physical activity and GDM

Supplementary Figure 2. A funnel plot showing the publication bias for dietary intervention and GDM

Supplementary Figure 3. A funnel plot showing the publication bias for combined lifestyle intervention and GDM

Supplementary Figure 4. A funnel plot showing the publication bias for metformin and GDM

Supplementary Figure 5. A funnel plot showing the publication bias for the effect of probiotics on preventing GDM

Supplementary Figure 6. A funnel plot showing the publication bias for the effect of myoinositol/inositol on preventing GDM

Supplementary Table 1. PICO/S of the study

Population	Intervention	Outcome	Limits
All women (of childbearing age)	Interventions:<br>• Diet<br>• Exercise<br>• Behavioral<br>• Lifestyle<br>• Combined (diet, behavioral, exercise)<br>• Metformin<br>• Supplementation<br><br>Control:<br>• Usual care<br>• Placebo<br>• Minimal intervention (e.g. not more than 1 session a year	Primary<br>Gestational diabetes<br><br>Secondary<br>Gestational weight gain	Randomised or non-randomised controlled trials<br><br>Language: English<br><br>Years of publication: all years

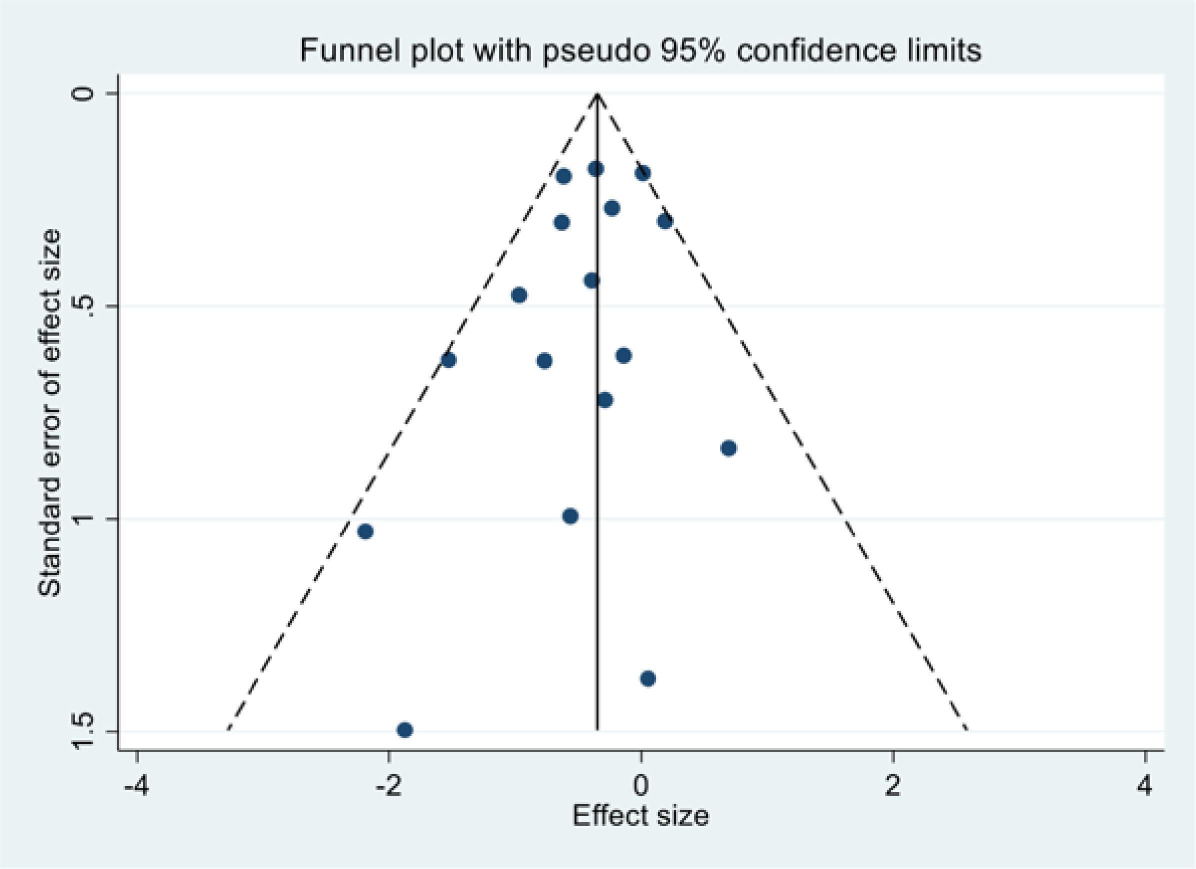

Supplementary Figure 1. A funnel plot of publication bias for studies on physical activity interventions.

Legend. The figure illustrates the publication bias of studies included to examine the effect of physical activity interventions on reducing the risk of GDM. The dots in the funnel plot represent the studies included in the analysis to examine publication bias. The distribution of the studies on the left and right of the solid vertical line (pooled effect size) seems symmetric, suggesting the absence of publication bias.

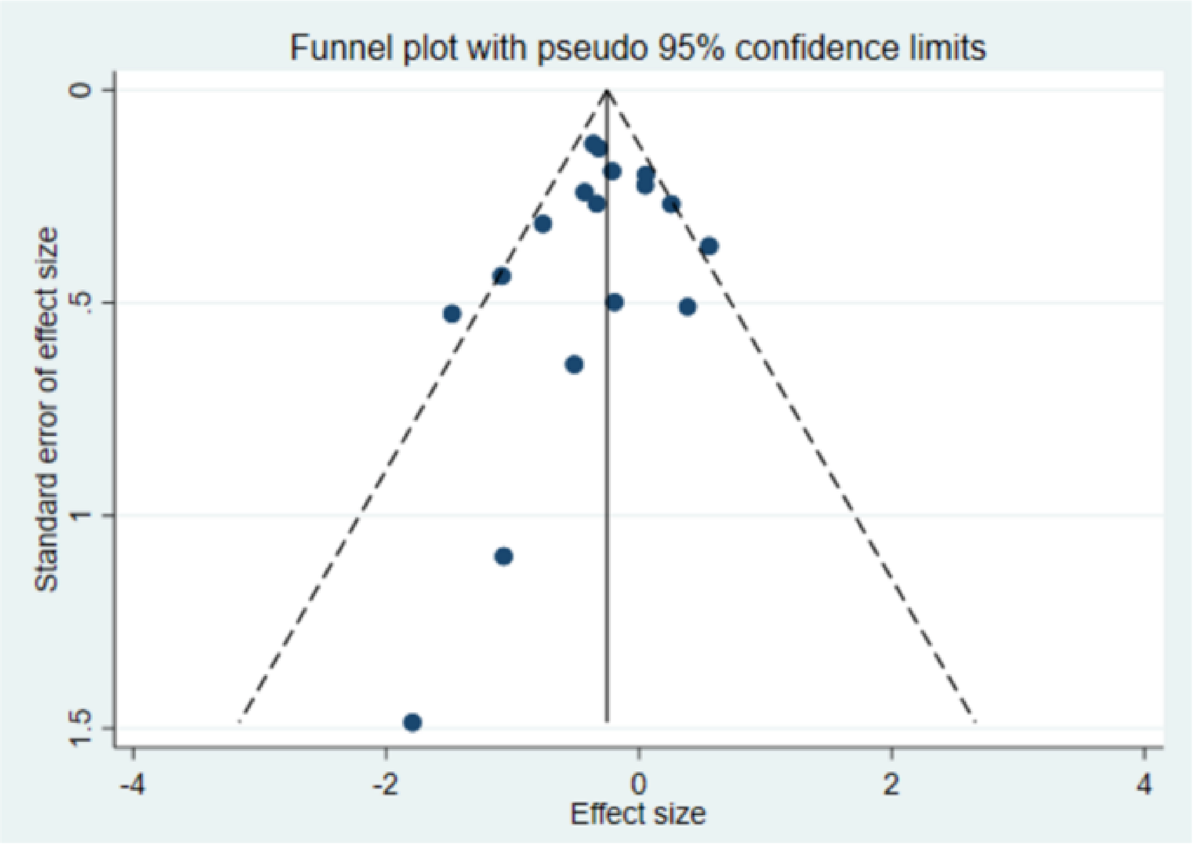

Supplementary Figure 2. A funnel plot of publication bias for studies on dietary interventions.

Legend. The figure illustrates the publication bias of studies included to examine the effect of dietary interventions on reducing the risk of GDM. The dots in the funnel plot represent the studies included in the analysis to examine publication bias. The distribution of the studies on the left and right of the solid vertical line (pooled effect size), seems symmetric, suggesting the absence of publication bias.

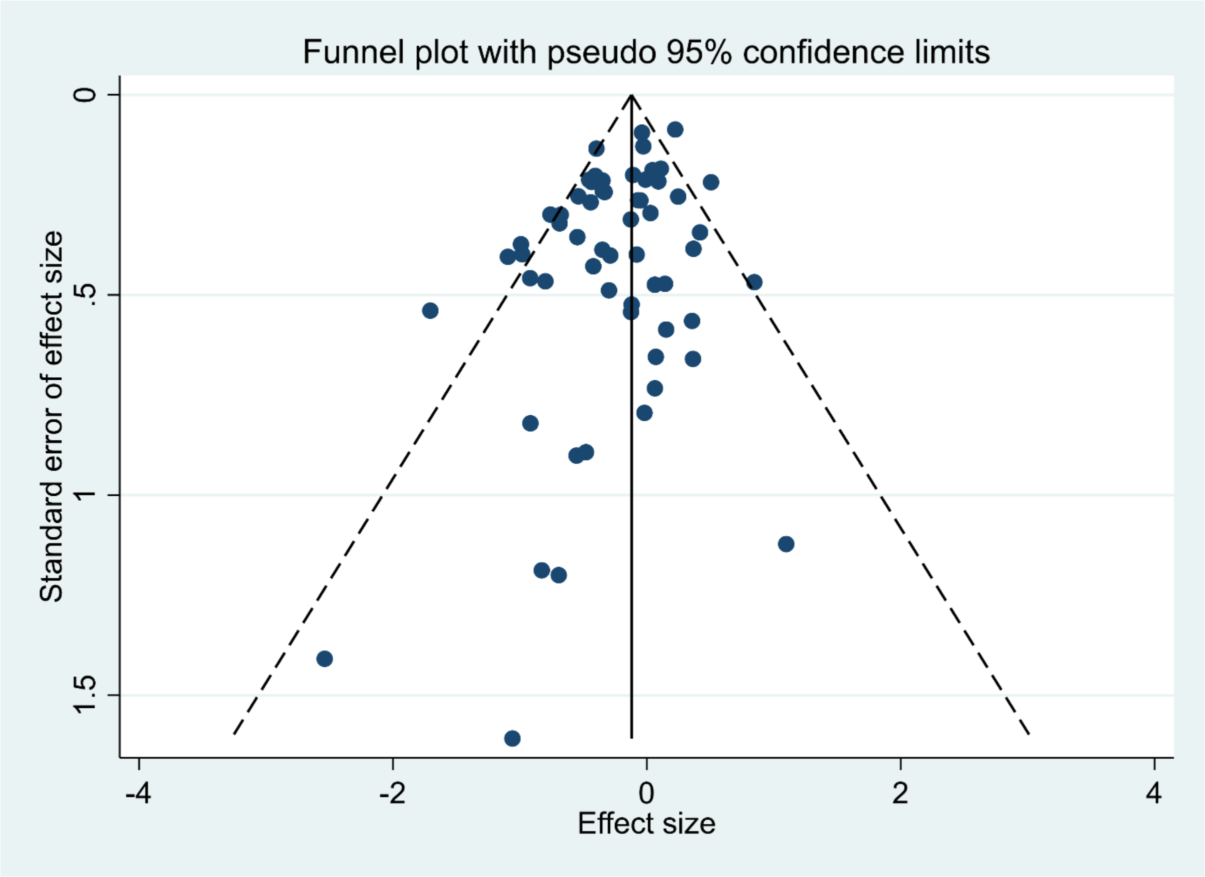

Supplementary Figure 3. A funnel plot of the publication bias for studies on combined physical activity and dietary interventions.

Legend. The figure illustrates the publication bias of studies included to examine the effect of combined physical activity and dietary interventions on reducing the risk of GDM. The dots in the funnel plot represent the studies included in the analysis to examine publication bias. The distribution of the studies on the left and right of the solid vertical line (pooled effect size) seems asymmetric, with more studies concentrated in the left bottom of the funnel, but not to the right bottom), suggesting the presence of publication bias.

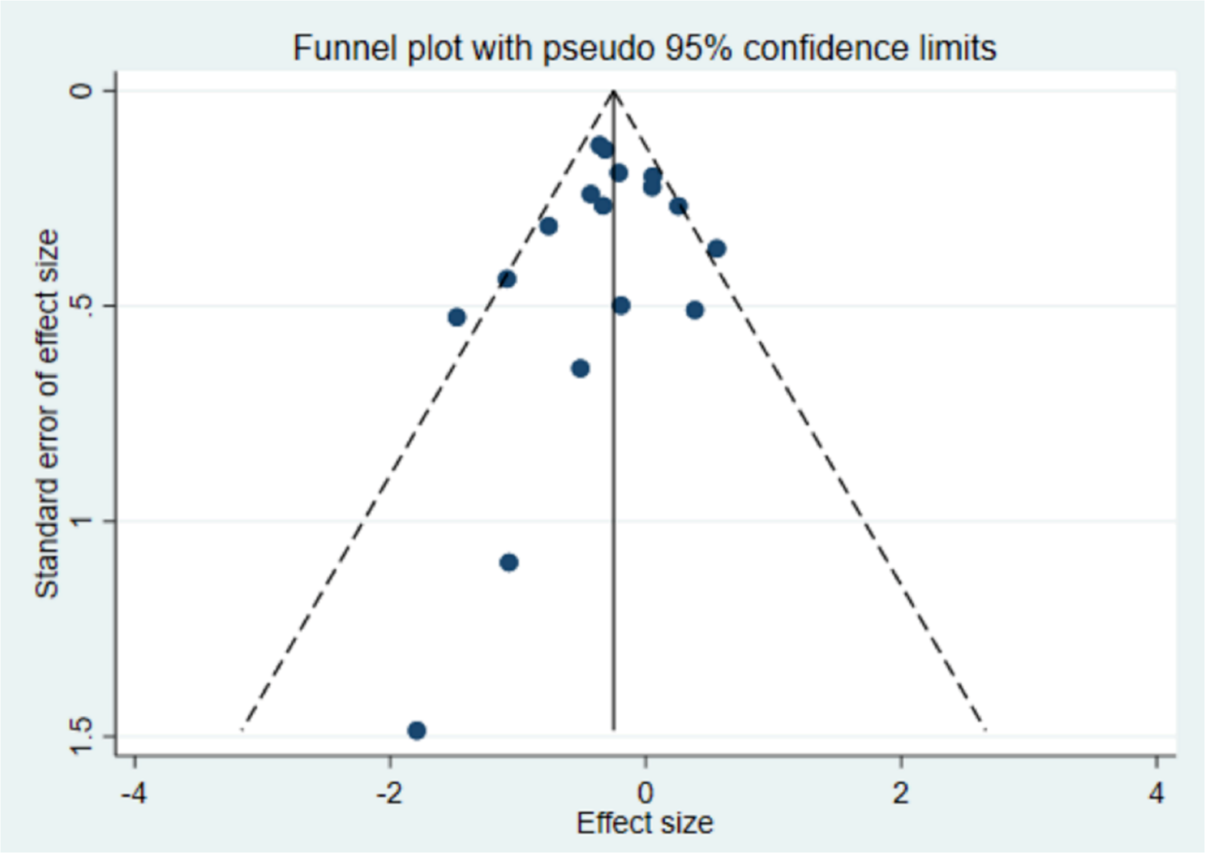

Supplementary Figure 4. A funnel plot of the publication bias for studies on metformin administration.

Legend. The figure illustrates the publication bias of studies included to examine the effect of metformin administration on reducing the risk of GDM. The dots in the funnel plot represent the studies included in the analysis to examine publication bias. The distribution of the studies on the left and right of the solid vertical line (pooled effect size) seems asymmetric, with more studies concentrated in the left bottom of the funnel, but not to the right bottom), suggesting the presence of publication bias.

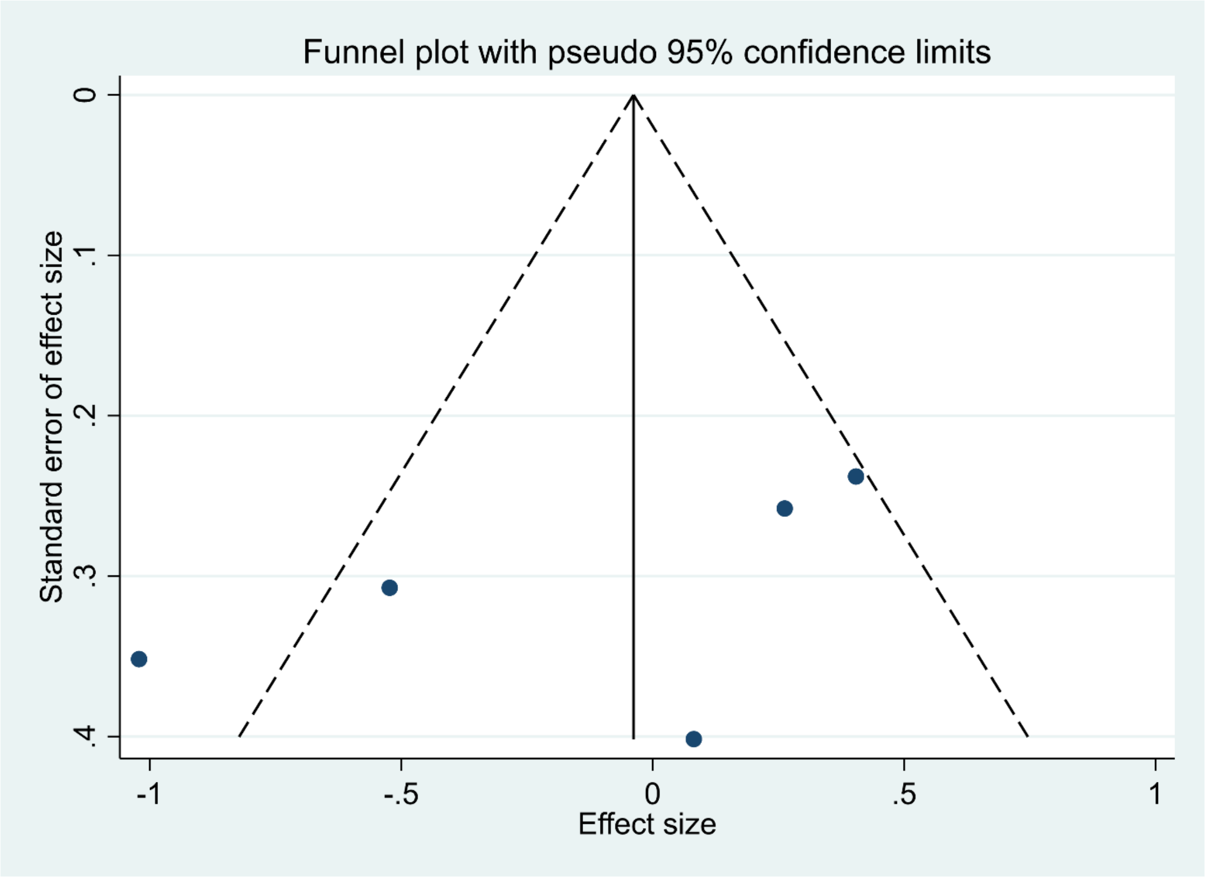

Supplementary Figure 5. A funnel plot of the publication bias for studies on probiotic supplementation.

Legend. The figure illustrates the publication bias of studies included to examine the effect of probiotic supplementation on reducing the risk of GDM. The dots in the funnel plot represent the studies included in the analysis to examine publication bias. The distribution of the studies on the left and right of the solid vertical line (pooled effect size), seems symmetric, suggesting the absence of publication bias.

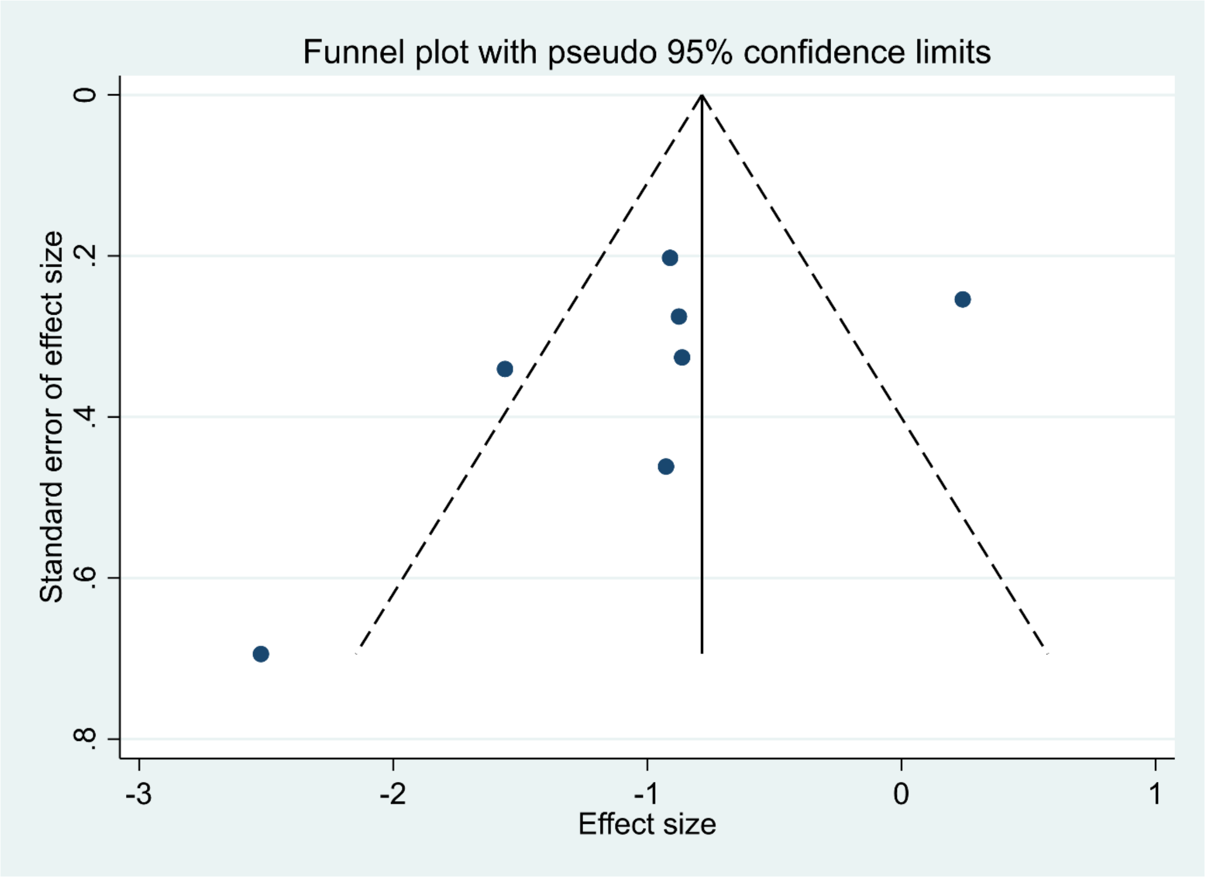

Supplementary Figure 6. A funnel plot of the publication bias for studies on myoinositol/inositol supplementation.

Legend. The figure illustrates the publication bias of studies included to examine the effect of myoinositol/inositol supplementation on reducing the risk of GDM. The dots in the funnel plot represent the studies included in the analysis to examine publication bias. The distribution of the studies on the left and right of the solid vertical line (pooled effect size), seems symmetric, suggesting the absence of publication bias.
