# Supplementary material for: Effective interventions in preventing gestational diabetes mellitus: A systematic review and meta-analysis
Source: Commun Med (Lond). 2024 Apr 20;4:75. doi: 10.1038/s43856-024-00491-1 (PMC11032369; doi:10.1038/s43856-024-00491-1)
Supplement: Supplementary file 2 — Description of Additional Supplementary File [file 43856_2024_491_MOESM2_ESM.docx]

**Description of Additional Supplementary File**

**File name:** Supplementary Data 1

**Description:** Databases used with the search strategy

**File name:** Supplementary Data 2

**Description:** Definition of intervention characteristics (TIDieR)

**File name:** Supplementary Data 3

**Description:** Characteristics of the included individual studies

**File name:** Supplementary Data 4

**Description:** Risk of bias assessment finding

**File name:** Supplementary Data 5

**Description:** Summary of certainty of evidence (GRADE)

**File name:** Supplementary Data 6

**Description:** Summary of studies on lifestyle intervention (diet-only, diet and physical activity combined, and physical activity only) by characteristics

**File name:** Supplementary Data 7

**Description:** Sub-group analysis of physical activity intervention by intervention characteristics

**File name:** Supplementary Data 8

**Description:** Summary of intervention characteristics for studies on the effect of metformin risk of GDM

**File name:** Supplementary Data 9

**Description:** Summary of intervention characteristics for studies on the effect of myoinositol/inositol and probiotics risk of GDM
