# Supplementary material for: Effective interventions in preventing gestational diabetes mellitus: A systematic review and meta-analysis
Source: Commun Med (Lond). 2024 Apr 20;4:75. doi: 10.1038/s43856-024-00491-1 (PMC11032369; doi:10.1038/s43856-024-00491-1)
Supplement: Supplementary file 10 — Supplementary Data 8 [file 43856_2024_491_MOESM10_ESM.docx]

**Supplementary Data 8. Summary of intervention characteristics for studies on the effect of metformin risk of GDM (N=13)**

| **Study, Year** | **Country** | **Intervention description - Material/tool;**  **Dose** | **Intervention description - Procedure** | **Control description** | **Tailoring (e.g. individualised goals)** | **Duration of active intervention (weeks)** | **Application of theoretical framework/model** | **Application of technologies (website, video, phone app, etc)** | **Adherence;**  **Attrition** |
| --- | --- | --- | --- | --- | --- | --- | --- | --- | --- |
| Adb El Hameed et al, 2011 | Egypt | Yes  1000-2000mg/day | Yes | No | No | Unspecified | No | NR | NR  0 |
| Ainuddin et al, 2015 | Pakistan | NR  1500mg/day | Yes | Yes | No | Unspecified | No | NR | Yes  0 |
| Chiswick et al, 2008 | UK | NR  500-2500mg/day | Yes | Yes | No | 20 | No | NR | Yes  42 |
| Dodd et al, 2018 | Australia | NR  500mg-2000mg/day | Yes | No | Yes | 28 | Yes | Yes | Yes  1.9 |
| Glueck et al, 2002b | USA | NR  2550mg/day | Yes | Yes | No | 23.2 | No | NR | Yes  29.6 |
| Glueck et al, 2002a | America | NR  2250mg/day | Yes | No | No | Unspecified | No | NR | Yes  0 |
| Jamal et al, 2012 | Iran | Yes  2000mg/dl | Yes | Yes | No | Unspecified | No | NR | NR  0 |
| Khattab et al, 2011 | Egypt | NR  1000-2000mg/day | Yes | Yes | Yes | 18 | No | NR | NR  0 |
| Lovvik et al, 2019 | Norway | NR  500-1000mg/day | Yes | Yes | No | Unspecified | No | NR | Yes  0 |
| Sales et al, 2018 | Brazil | NR  1000mg/day | Yes | No | No | 20 | No | NR | NR  0 |
| Syngelaki et al, 2016 | UK | Yes  1000-3000gm/day | Yes | Yes | No | Unspecified | No | NR | Yes  11 |
| Valdés et al, 2018 | Spain | Yes  1700mg/day | Yes | Yes | No | Unspecified | No | NR | NR  21 |
| Vanky et al, 2010 | Norway | Yes  2000mg/day | Yes | Yes | No | 36 | No | NR | Yes  9 |
