# Supplementary material for: Effective interventions in preventing gestational diabetes mellitus: A systematic review and meta-analysis
Source: Commun Med (Lond). 2024 Apr 20;4:75. doi: 10.1038/s43856-024-00491-1 (PMC11032369; doi:10.1038/s43856-024-00491-1)
Supplement: Supplementary file 13 — Reporting Summary [file 43856_2024_491_MOESM13_ESM.pdf]

nature portfolio

Corresponding author(s): DBPR: COMMSMED-23-0528

Last updated by author(s): 21/03/2024

Reporting Summary

Nature Portfolio wishes to improve the reproducibility of the work that we publish. This form provides structure for consistency and transparency in reporting. For further information on Nature Portfolio policies, see our Editorial Policies and the Editorial Policy Checklist.

Statistics

For all statistical analyses, confirm that the following items are present in the figure legend, table legend, main text, or Methods section.

n/a	Confirmed	
☒	☐	The exact sample size (*n*) for each experimental group/condition, given as a discrete number and unit of measurement
☒	☐	A statement on whether measurements were taken from distinct samples or whether the same sample was measured repeatedly
☐	☒	The statistical test(s) used AND whether they are one- or two-sided<br>*Only common tests should be described solely by name; describe more complex techniques in the Methods section.*
☐	☒	A description of all covariates tested
☒	☐	A description of any assumptions or corrections, such as tests of normality and adjustment for multiple comparisons
☐	☒	A full description of the statistical parameters including central tendency (e.g. means) or other basic estimates (e.g. regression coefficient) AND variation (e.g. standard deviation) or associated estimates of uncertainty (e.g. confidence intervals)
☐	☒	For null hypothesis testing, the test statistic (e.g. *F*, *t*, *r*) with confidence intervals, effect sizes, degrees of freedom and *P* value noted<br>*Give P values as exact values whenever suitable.*
☒	☐	For Bayesian analysis, information on the choice of priors and Markov chain Monte Carlo settings
☒	☐	For hierarchical and complex designs, identification of the appropriate level for tests and full reporting of outcomes
☐	☒	Estimates of effect sizes (e.g. Cohen's *d*, Pearson's *r*), indicating how they were calculated

Our web collection on statistics for biologists contains articles on many of the points above.

Software and code

Policy information about availability of computer code

Data collection	As the study is systematic review and meta-analysis, we have extracted data from the eligible original studies.
Data analysis	The data were analysed using STATA/SE TM Version 17. Risk ratios (RR) and 95% confidence intervals (CI) were pooled using the random-effects model by applying the DerSimonian and Laird estimator. Heterogeneity was examined by the I2 statistic. Sensitivity analysis was carried out by excluding non-RCTs assuming the study design could impact the risk estimate due to lack of randomization. Subgroup analysis by intervention characteristics was performed. A funnel plot and Egger's test were used to examine publication bias. Asymmetry of the funnel plots and significant Egger's test (p<0.05) suggest publication bias.

For manuscripts utilizing custom algorithms or software that are central to the research but not yet described in published literature, software must be made available to editors and reviewers. We strongly encourage code deposition in a community repository (e.g. GitHub). See the Nature Portfolio guidelines for submitting code & software for further information.

Data

Policy information about availability of data

All manuscripts must include a data availability statement. This statement should provide the following information, where applicable:

- Accession codes, unique identifiers, or web links for publicly available datasets
- A description of any restrictions on data availability
- For clinical datasets or third party data, please ensure that the statement adheres to our policy

The data that support the findings of this study are available from the authors upon reasonable request and with permission from the Eastern Health Clinical School, Monash University, Australia

Human research participants

Policy information about studies involving human research participants and Sex and Gender in Research.

Reporting on sex and gender	Our study is a systematic review and meta-analysis involving studies conducted in women. All studies upon review used the term 'women' and we used the same terminology to be consistent. But we acknowledge that not all individuals who experienced pregnancy may not self-identified as 'women' at all times over their life courses.
Population characteristics	This systematic review included studies in preconception, pregnancy and postpartum periods.
Recruitment	This is a systematic review and meta-analysis, and no recruitment was conducted.
Ethics oversight	This is a systematic review and meta-analysis, and no ethics approval was needed.

Note that full information on the approval of the study protocol must also be provided in the manuscript.

Field-specific reporting

Please select the one below that is the best fit for your research. If you are not sure, read the appropriate sections before making your selection.

☒ Life sciences ☐ Behavioural & social sciences ☐ Ecological, evolutionary & environmental sciences

For a reference copy of the document with all sections, see nature.com/documents/nr-reporting-summary-flat.pdf

Life sciences study design

All studies must disclose on these points even when the disclosure is negative.

Sample size	As our study is systematic review and meta-analysis, sample size determination was not done.
Data exclusions	Exclusion criteria was established according to the protocol, in terms of relevance to the research question.
Replication	All data should be reproducible. Methods are as explicitly described in the published protocol
Randomization	No applicable
Blinding	Not applicable

Reporting for specific materials, systems and methods

We require information from authors about some types of materials, experimental systems and methods used in many studies. Here, indicate whether each material, system or method listed is relevant to your study. If you are not sure if a list item applies to your research, read the appropriate section before selecting a response.

Materials & experimental systems

n/a	Involved in the study
☒	☐ Antibodies
☒	☐ Eukaryotic cell lines
☒	☐ Palaeontology and archaeology
☒	☐ Animals and other organisms
☒	☐ Clinical data
☒	☐ Dual use research of concern

Methods

n/a	Involved in the study
☒	☐ ChIP-seq
☒	☐ Flow cytometry
☒	☐ MRI-based neuroimaging
